# Proteomic analysis of affinity-purified extracellular proteasomes reveals exclusively 20S complexes

**DOI:** 10.18632/oncotarget.22230

**Published:** 2017-11-01

**Authors:** Valentina A. Kulichkova, Tatiana O. Artamonova, Olga G. Lyublinskaya, Mikhail A. Khodorkovskii, Alexey N. Tomilin, Anna S. Tsimokha

**Affiliations:** ^1^ Institute of Cytology, Russian Academy of Sciences, St-Petersburg 194064, Russia; ^2^ Institute of Nanobiotechnologies, Peter the Great St-Petersburg Polytechnic University, St-Petersburg 195251, Russia; ^3^ Institute of Translational Biomedicine, St-Petersburg State University, St-Petersburg 199034, Russia

**Keywords:** extracellular proteasome, human leukemia K562 cells, proteasome interacting protein (PIP), affinity purification, mass spectrometry

## Abstract

Proteasome-mediated proteolysis is important for many basic cellular processes. In addition to their functions in the cell, proteasomes have been found in physiological fluids of both healthy and diseased humans including cancer patients. Higher levels of these proteasomes are associated with higher cancer burden and stage. The etiology and functions of these proteasomes, referred to as circulating, plasmatic, or extracellular proteasomes (ex-PSs), are unclear. Here we show that human cancer cell lines, as well as human endometrium-derived mesenchymal stem cells (hMESCs), release proteasome complexes into culture medium (CM). To define ex-PS composition, we have affinity purified them from CM conditioned by human leukemia cell line K562. Using matrix-assisted laser desorption/ionization (MALDI) Fourier transform ion cyclotron resonance (FT-ICR) mass spectrometry (MS), we have identified core 20S proteasome subunits and a set of 15 proteasome-interacting proteins (PIPs), all previously described as exosome cargo proteins. Three of them, PPIase A, aldolase A, and transferrin, have never been reported as PIPs. The study provides compelling arguments that ex-PSs do not contain 19S or PA200 regulatory particles and are represented exclusively by the 20S complex.

## INTRODUCTION

Proteasomes are responsible for the majority of non-lysosomal protein degradation in eukaryotic cells. Accordingly, they are involved in many important biological processes such as cell cycle progression, apoptosis, stress response and regulation of the immune and inflammatory responses [1, 2]. Proteasomes are localized in both the cell cytoplasm and the nucleus [3], and can be released into the extracellular space [4, 5].

The term proteasome refers to several complexes with multiple functions and specificities in a single eukaryotic cell. The 26S proteasome is an ATP-dependent protease that selectively degrades various cellular proteins carrying specific degradation signals such as a polyubiquitin chain [6, 7]. The 26S proteasome is composed of the 20S core particle (20S CP) capped by the 19S regulatory particle (19S RP) at one or both ends. The 19S RP contains ATPases and a recognition sites for polyubiquitinated proteins which are subject to degradation.

The eukaryotic 20S CP consists of 14 subunits which are arranged in four stacked rings, comprising two outer α-rings and two inner β-rings [8]. The α- and β-rings are each composed of seven α- and β-subunits, respectively. Three of the seven β-subunits, β1, β2, and β5, are catalytically active and responsible for the proteolytic activity of the proteasome. The 20S CP degrades small peptides and fully unfolded proteins in an ATP- and ubiquitin-independent manner. The proteolytic activities of proteasomes are described as caspase-, trypsin-, and chymotrypsin-like [9]. A second proteasome subpopulation with distinct proteolytic activities is a result of replacement of the constitutive catalytic subunits β1, β2, and β5 with inducible subunits β1i, β2i, and β5i, and this replacement occurs upon induction with INF-γ. The formation of an inducible 20S CP (immunoproteasome) results in the efficient generation of peptides that are presented by MHC class I complexes. In addition to the standard proteasome and immunoproteasome, several tissue-specific 20S species have been identified, including the thymoproteasome and spermatoproteasome subunits [10]. The 20S CP in the thymus predominantly contains the specific β5t subunit and are thought to be primarily associated with antigenic peptide generation for positive selection of T lymphocytes [11], while the mammalian testis-specific proteasomes contain alternative α4s subunits, implicated together with the nuclear PA200 in the degradation of acetylated histones during spermatogenesis and DNA repair [12, 13]. Recently, the formation of an α4-α4 proteasome isoform was reported in human cells [14]. Cells primed to assemble this proteasome isoform species exhibit enhanced resistance to toxic metal ions.

In addition, the 20S CP can be associated with other regulatory complexes, including PA200 and those of PA28 family: PA28α/β and PA28γ[15]. The 20S CP in complex with these regulators performs ubiquitin-independent proteolysis. The PA28α/β activator is implicated in the processing of MHC class I antigens [16]. The activator complex PA28γ possibly plays a role in cell division and apoptosis [17]. The single-chain protein PA200 is thought to play a role in DNA repair [18] and spermatogenesis [19]. In that way, the heterogeneity in the proteasome subunit composition and their functional diversity allows cells to meet their intracellular requirements, including the response to stress or other stimuli.

Importantly, proteasomes have also been identified in the extracellular space, such as blood plasma, the cerebrospinal and alveolar fluids [4, 20], as well as in culture medium conditioned by some human cell lines [21]. The proteasomes which have been detected in normal human blood plasma are variously referred to as “circulating proteasomes” (c-proteasomes), “plasma-proteasomes” (p-proteasomes), or ex-PSs (this paper). It was observed that concentrations of ex-PSs in blood plasma from patients with hematologic malignancies, multiple myeloma, acute and chronic lymphatic leukemia, solid tumors, autoimmune diseases, sepsis or trauma were substantially higher than in the plasma of healthy patients [4]. Therefore, ex-PSs are regarded as potential diagnostic and even prognostic biomarkers of various diseases [22].

The origin of ex-PSs remains largely unknown, neither their function nor precise subunit composition. As shown by electron microscopy, plasma from healthy patients also contained 20S CPs, which, after purification, were enzymatically active and had subunit patterns different from those in blood cells [23]. Moreover, we previously demonstrated that purified ex-PSs from culture medium (CM) comprised the 20S proteins and low amounts of the 19S proteins [24]. However, a constant supply of ATP is necessary to maintain 26S proteasome in an intact state, as ATP withdrawal causes rapid 26S proteasome dissociation into 20S CP and 19S RPs. Because ATP is not a component of standard cellular media, 19S RP or alternative regulators could be lost due to dissociation 26S proteasome or hybrid complexes during purification of ex-PSs. In this article, however, we show that neither 19S RP subunits nor PA200 protein are present CM conditioned by the human leukemic cell line K562 and supplemented with ATP. We also define exact composition of these ex-PSs, using a combination of biochemical, affinity purification approaches, matrix-assisted laser desorption/ionization (MALDI) Fourier transform ion cyclotron resonance (FT-ICR) mass spectrometry (MS), and database search. Our data show that both tumor and normal cells release ex-PSs, and the population of these ex-PSs does not contain 19S RP or PA200 regulator but consists exclusively of 20S CPs.

## RESULTS

The molecular characterization of extracellular proteasomes (ex-PSs) has been a challenging task, largely because of the difficulty in purifying these complexes. In this study, we have made use of the previously reported tandem affinity-tagged β7 subunit [25] to extract ex-PSs in their native state from CM.

### Optimization of CM for ex-PS purification

**Figure 1:**
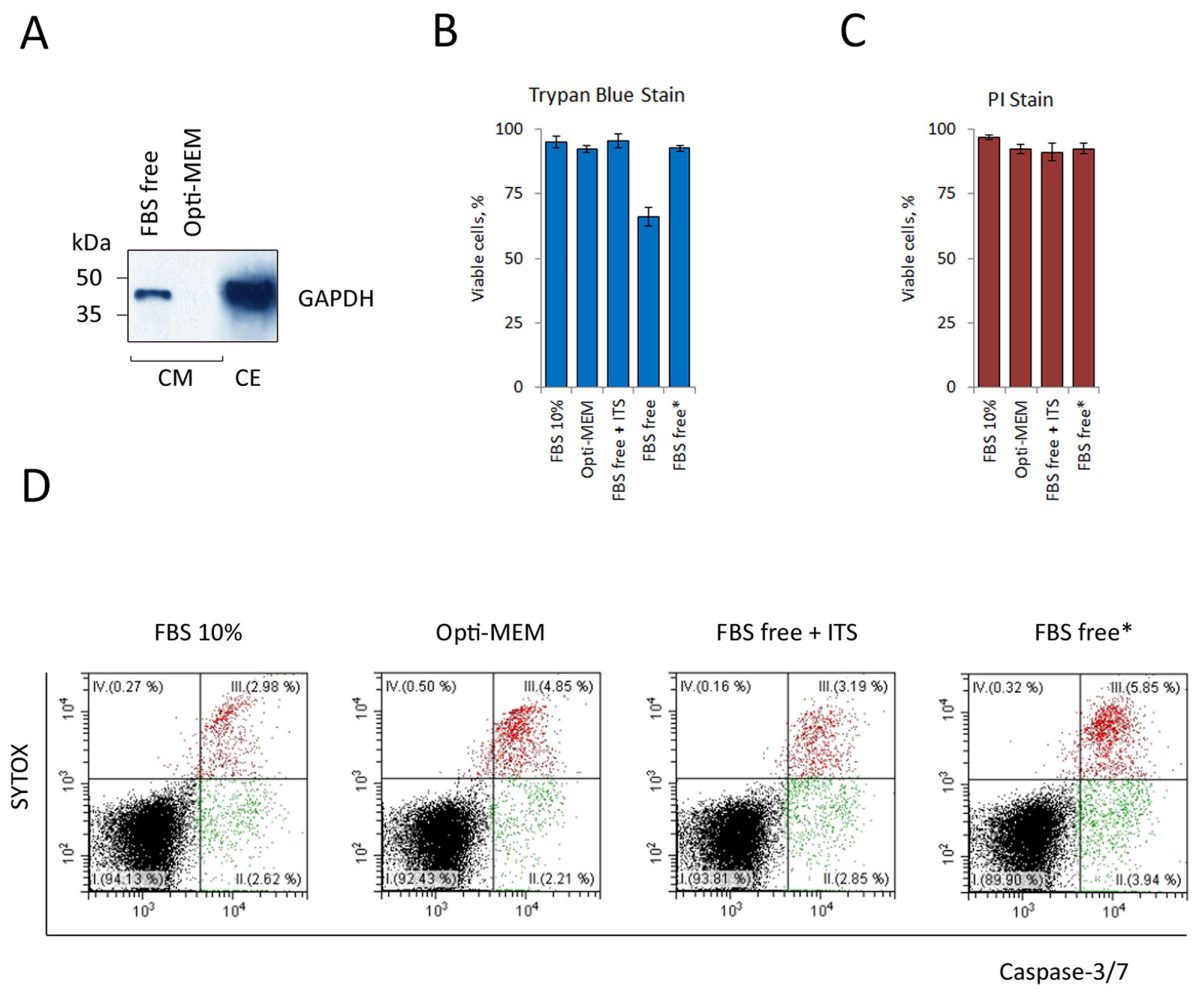
Optimization of serum-free culture medium for a continuous K562 cells cultivation without cell death **(A)** 15 μg whole-cell extract (CE) and culture medium, conditioned by 15×10^6^ cells (CM) were subjected to SDS-PAGE and analyzed by Western blotting for their content of the cellular protein GAPDH. Western blots are representative of three independent experiments. Representative Western blot showing the presence of GAPDH in the CM after overnight cultivation in culture medium (RPMI-1640) without FBS (FBS free). Opti-MEM® Reduced Serum Medium (Invitrogen) rescued K562 cells from serum-free cell death. Relative cell viability was assessed by the trypan blue dye exclusion method **(B)** and the propidium iodide (PI) flow cytometric assay **(C)**. Results are presented as percentage cell viability (means ± standard deviation, n = 3). Opti-MEM, FBS free medium with minimal growth factors (FBS free + ITS) and two-fold reduction in cell seeding density (FBS free^*^) rescued K562 cells from serum-free cell death. **(D)** Serum-free induced apoptosis in K562 cells as assessed by double staining with CellEvent caspase-3/7 green detection reagent and the vital dye SYTOX. Data of one representative experiment are shown. The experiment was repeated three times with similar results.

Most MS-based proteomics approaches developed to study structural topologies of proteasome complexes start with the preparation and purification of proteasomes [26]. We have, however, encountered several problems during the purification of proteasomes from CM. One of the problems was the low concentration of proteasomes in the CM. This problem has been resolved by means of concentrating proteins from CM using ultracentrifugal filters (Amicon). Secondly, the presence of a large amount of protein in complete serum-containing culture medium (BSA, in particular) further complicates proteomic analysis of ex-PSs, as the latter remain minor components even after CM concentration. To circumvent this problem we produced CM under serum-free conditions. RPMI-1640 medium with minimal growth factors, such as insulin, transferrin, and selenium (ITS) [27] or special serum-free media (e.g. Opti-MEM® Reduced Serum Medium, Invitrogen) were successfully applied for most cell lines. We report an optimization of serum-free culture medium for cultivation of K562 cells for ex-PS preparation (Figure 1). Predictably, culturing overnight in serum-free media without supplements (1×10^6^ cells/1 ml media) led to partial cell death manifested as the appearance of intracellular protein GAPDH in the CM (Figure 1A) and a decrease of cell viability (Figure 1B). Replacing the serum-free medium with Opti-MEM, supplementing the minimal RPMI-1640 with ITS, or initial seeding of the cells at a lower density (0.5×10^6^ cells/2 ml media) all increased the viability of K562 cells (Figure 1B and Supplementary Figure 1). We confirmed these conclusions by PI staining (Figure 1C) and the caspase 3/7 activation assay (Figure 1D).

### ex-PSs are released by various tumor and primary cells

**Figure 2:**
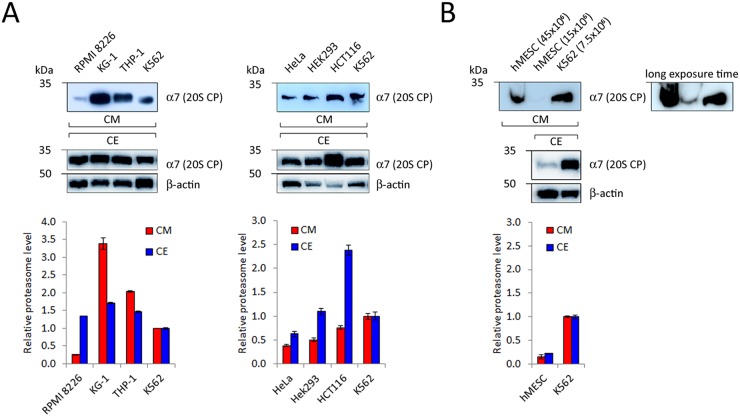
Detection of 20S proteasome subunits in CM conditioned by different human hematopoietic **(A, left panel)** and epithelial cancer cells **(A, right panel)**, as well as by human mesenchymal stem cells **(B, hMESC)**. Samples of CM, conditioned by 15×10^6^ cells, and 15μg of CE were subjected to SDS-PAGE and Western blotted. Representative blots of three independent experiments are shown. Densitometric analysis was performed and proteasome levels in CM and CE of various cell types are shown as relative intensities in comparison with K562 cells (means ± standard deviation, n = 3). Actin served as a loading control and was used for normalization for CE samples. CM samples were normalized by equal number of cells.

Previously, we have shown that proteasomes are detectable in CM conditioned by human tumor cell lines [21, 28]. It is known that expression of proteasomes is overall enhanced (compared to normal cells) but varies amongst different tumor cells [29–36]. Furthermore, a positive correlation between levels of PS and ex-PS in CE and CM can be expected. To address these points, we have analyzed proteasome levels in CE and CM samples conditioned by equal numbers of different cancer cells (15×10^6^), such as human hematopoietic and epithelial cancer cell lines (Figure 2A). All tested cells released ex-PSs into the CM, however, to our surprise, no positive correlation between the PS and ex-PS levels was found (Figure 2A).

Tumor cells are known to display a characteristic set of features that distinguish them from normal cells and the release of ex-PSs could be one such feature. To address this possibility, we compared CM from primary cultures of human mesenchymal stem cells (hMESCs) and K562 cells by Western blotting, using antibodies to a 20S CP subunit. Proteasome signal was not present in CM from hMESCs but readily detectable in CM harvested from an equal number of K562 cells (Supplementary Figure 2). We were able to detect ex-PSs in CM harvested from hMESCs only after scaling up CM volumes and cell numbers 2- to 6-fold (Figure 2B). Our data suggest that ex-PSs are in fact produced by the cultured primary hMESCs, albeit at levels significantly lower than by cancer cells.

### Analysis of proteasomal activity and 20S subunit composition in CM

**Figure 3:**
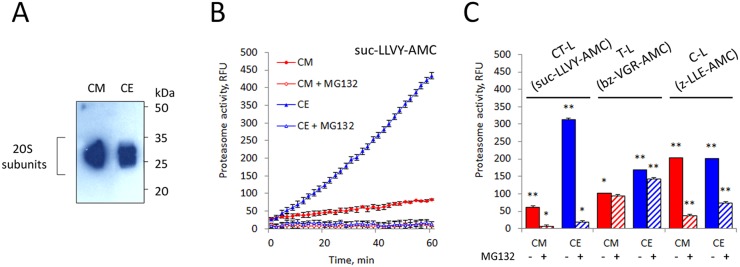
Analysis of proteasomal activity and subunit composition in K562 cell conditioned medium (CM) **(A)** 10 μg whole cell extract (CE) and culture medium conditioned by 10×10^6^ cells (CM) were subjected to SDS-PAGE and analyzed by Western blotting for their content of the 20S proteasome subunits. Representative blots of three independent experiments are shown. **(B)** CM was tested for proteasomal chymotrypsin-like activity with the substrate suc-LLVY-AMC. CM showed six-fold lower suc-LLVY-AMC hydrolysis activity than CE. **(C)** The three different proteolytic activities in CM were measured by using fluorogenic peptide substrates: suc-LLVY-AMC (for chymotrypsin-like activity), bz-VGR-AMC (for trypsin-like) and z-LLE-AMC (caspase-like), in the presence and absence of the inhibitor MG132. Values shown are mean ± standard deviation from three independent experiments (^*^p<0.01, ^**^p<0.001).

We have monitored the content of proteasome in the concentrated CM and whole cell extract (CE) of K562 cells by Western blot with an antibody to anti-proteasome subunits and by the analysis of their proteolytic activity. Remarkably, chymotrypsin (CT)-like proteasome activity in CM is lower compared to that in CE despite equal proteasome concentrations in the samples (Figure 3).

### Proteasome subtypes in CM

The non-ATPase subunit Rpn11 tagged at its C-terminus with the HTBH tag was used to purify the ex-PSs. This subunit has been shown to be a successful affinity bait for purifying human proteasomes without affecting their biological activity [37]. The HTBH tag consists of two hexahistidine runs (H), a tobacco etch virus (TEV) cleavage site (T), and a bacterially derived peptide that is subject to biotinylation *in vivo* (B). The HTBH tag allows two-step purification of proteasomes from mammalian cells via high-affinity streptavidin binding and TEV cleavage-mediated elution [37]. This strategy however did not allow us to purify ex-PSs (Supplementary Figure 3). However, we have previously revealed by iTRAQ quantitative proteomics that ex-PSs are deficient in 19S subunits [24], so the 20S subunit β7 tagged at its C-terminus with HTBH [25] was used instead of Rpn11 to purify ex-PSs.

**Figure 4:**
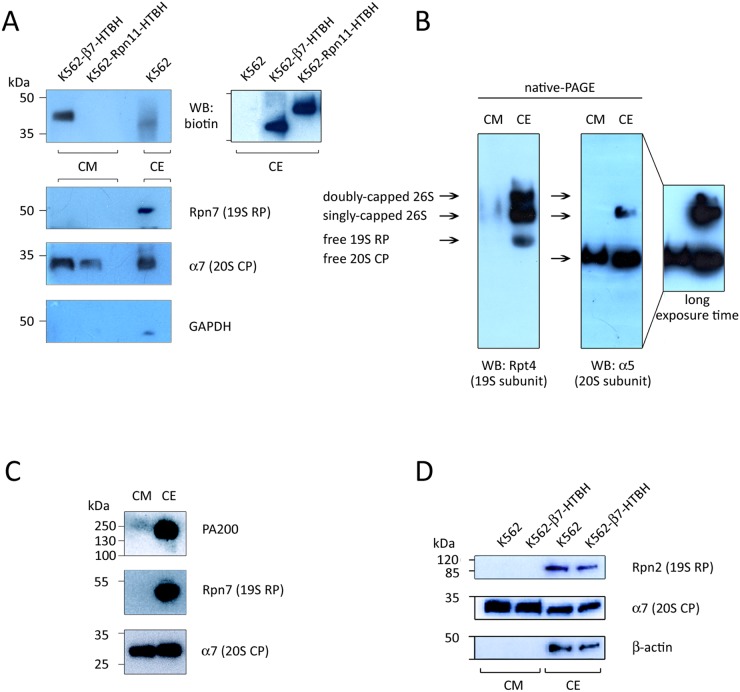
Immunochemical detection of subunits of 20S proteasomes, 19S regulatory particles (19S RP) and PA200 in the conditioned medium (CM) **(A and D)** Material of CM, conditioned by wild type K562, β7-HTBH K562 and K562-Rpn11-HTBH K562 cells (10^7^ cells), and whole cell extract (CE, 10 μg) was subjected to SDS-PAGE and Western blotting. Upper panel shows the HTBH-tagged proteasome subunit. Levels of cell death were controlled by Western blotting with an antibody to GAPDH (and actin). (B) Whole cell extract (CE, 20 μg) and CM (conditioned by 20×10^6^ cells) were subjected to native PAGE and Western blotted. (C) CE (10 μg) and CM (conditioned by 10×10^6^ cells) were subjected to SDS-PAGE and Western blotted with antibodies against proteins of 20S, 19S and PA200 proteasome complexes.

CM conditioned by the K562 cell line stably expressing either the Rpn11-HTBH or β7-HTBH were analysed by SDS-PAGE/Western blotting, using antibodies to biotin, GAPDH, and proteasome subunits (Figure 4A). As expected, we did not observe Rpn11-HTBH protein in the CM conditioned by the Rpn11-HTBH K562 cells, however, this tagged subunit was readily detected in the CE of these cells (Figure 4A, upper right panel). The 20S CP subunit α7 was seen in the CM from K562 cell lines expressing either Rpn11-HTBH or the β7-HTBH. Importantly, neither the 19S RP subunit Rpn7 nor GAPDH were present in CM conditioned by the both K562 cell lines. This observation argues against cell damage as a source of ex-PSs.

Using native PAGE/Western blot analysis of the 19S and 20S subunits, we found only 20S CPs in the CM (Figure 4B). This is in contrast to PS complexes in CE, which are represented by four forms, corresponding to doubly- and singly-capped 26S proteasomes, as well as by free 19S RPs and 20S CPs (Figure 4B).

We also performed Western blot analysis of CM using antibodies against the 20S CP subunit α7, the 19S RP subunit Rpn7 and the alternative regulator PA200. All these PS subunits were detected in the CE, but only the α7 subunit was observed in the CM (Figure 4C). Again, neither the 19S RP subunit Rpn7 nor PA200 were found in the CM.

In addition, results of Western blotting analysis of CM from wild-type and β7-HTBH K562 cells showed that HTBH-tagging of 20S CPs did not inhibit their release by the cells (Figure 4D): this allowed subsequent affinity purification of ex-PSs, as described below.

### ex-PS purification

**Figure 5:**
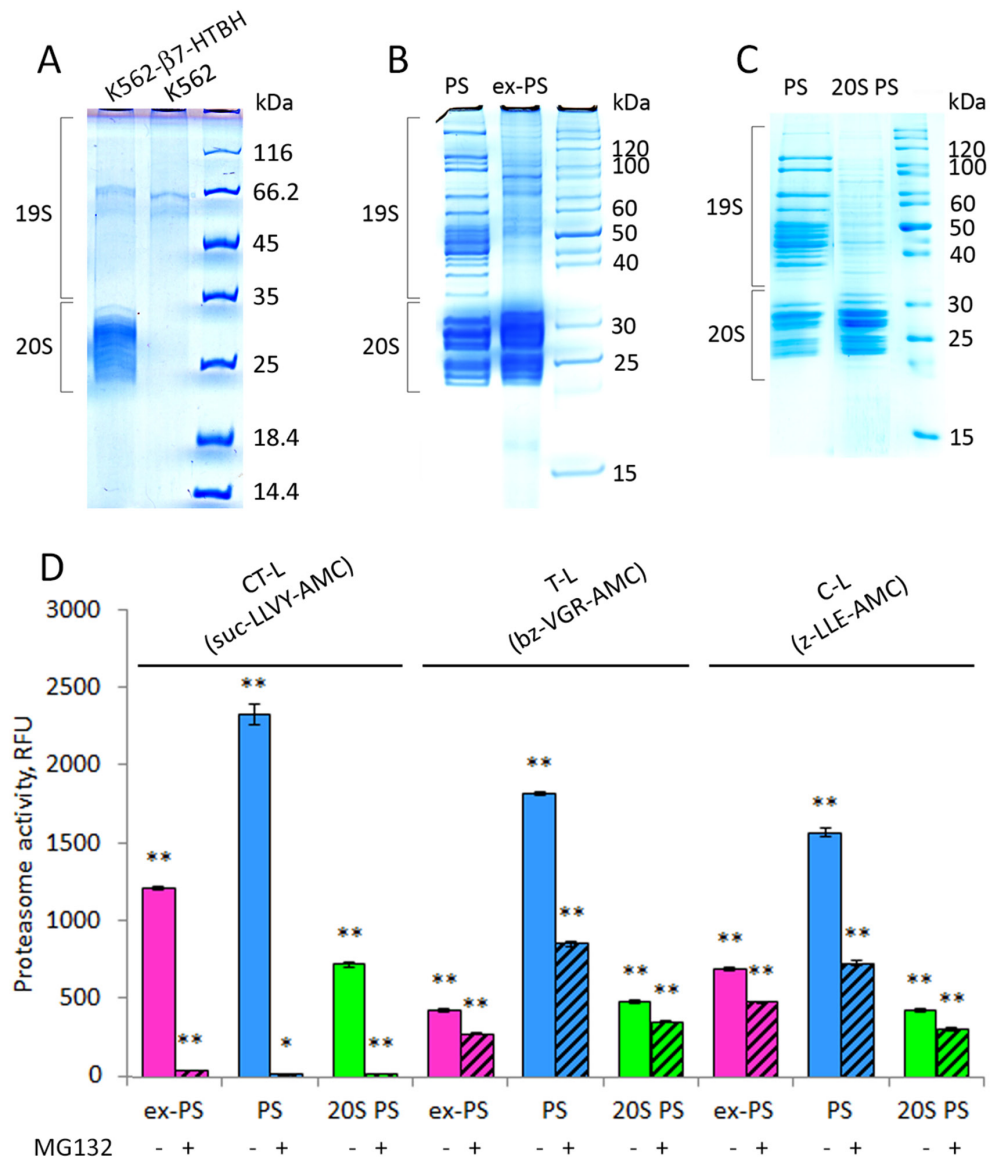
Affinity-purified proteasomes from conditioned medium (CM) and β7-HTBH K562 cells preserve chymotrypsin-like (CT-L) peptidase activity but decrease trypsin- and caspase-like (T-L and C-L) activities **(A)** 1D SDS-PAGE patterns of the purified samples from CM of control K562 cells and stable cell line expressing the β7-HTBH. **(B)** Proteins from affinity-purified cellular (PS) and extracellular (ex-PS) proteasomes (15 μg) were separated by SDS-PAGE, visualized with Coomassie Blue. Positions of 19S and 20S subcomplexes in the gel are shown. **(C)** Affinity-purified PS were dissociated into 20S (20S PS) and 19S subcomplexes by 1M NaCl treatment and then separated by SDS-PAGE followed by staining with Coomassie Blue. **(D)** Chymotrypsin- (CT-), trypsin- (T-), and caspase- (C-) like activities of ex-PS (1 μg) in comparison with PS in the presence or absence of proteasome inhibitor MG132 were determined by fluorometric quantification of the substrates Suc-LLVY-AMC, Ac-RLR-AMC and Z-LLE-AMC, using 380 nm excitation/440 nm emission, respectively. The results are given as Relative fluorescence units. Values shown are mean ± standard deviation from three independent experiments (^*^p<0.01, ^**^p<0.001).

The combination of affinity purification with mass spectrometry (MS) analysis has become the conventional method of choice for protein complex characterization, including proteasomes [24]. In order to identify as many proteasome components present in the CM as possible, large amounts of CM (0.5-1 L) were conditioned by the β7-HTBH K562 cells [25] and concentrated (approximately 100-fold) prior to affinity purification of ex-PSs. Approximately 200×10^6^ of β7-HTBH K562 cells released not more than 7 μg of tagged ex-PSs overnight. The purified samples from CM conditioned by the β7-HTBH K562 cells or untagged K562 cells (control) were separated by SDS-PAGE (Figure 5A). We analyzed the similar regions of the gel that were then treated with trypsin. The generated peptides were extracted, spotted onto a MALDI target plate and analyzed (Supplementary Figure 4). Only keratins, known contaminants of MS samples have been identified (Supplementary Table 1). Thus the purification of the ex-PSs using tagged subunit β7 appears to be highly specific. The purity of ex-PS preparations was verified by SDS-PAGE which, as expected, revealed a lack of protein bands corresponding to 19S subunits (Figure 5B).

### Activity of the purified ex-PSs

Next, we assessed the affinity-purified ex-PSs for CT-, trypsin(T)-, and caspase(C)-like activities, using a fluorogenic peptide substrate assay (Figure 5D). The fluorescence measured in the presence of the proteasome inhibitor MG132 was used as a control for the specificity of proteasomal activity. In spite of comparable amounts of ex-PSs and PSs, the CT-, T- and C-like peptidase activities of ex-PSs were about 50%, 15% and 25% of PSs, respectively (Figure 5D). Importantly, CT-like activities of PSs and ex-PSs were comparable and more than 95% inhibition of this activity was observed in the presence of MG132. Interestingly, the CT-like activity of proteasome in CM was about 6 times lower than in CE (Figure 3C), however, it increased to a normal level following the affinity purification procedure. Thus, the CT-like activity of purified ex-PSs was only 50% lower than that of PSs (Figure 5D). This observation is consistent with the previous observation that CT-like proteasomal activity in CM might be inhibited, likely by an unknown protein reversibly associated with the ex-PSs. Alternatively, it could be that ex-PSs do not contain 19S RPs, in contrast to the proteasome population in the cell (Figure 5D), and the activities of 20S complexes have been demonstrated to be significantly lower than the activities of 26S proteasomes [38, 39]. Therefore, we compared the peptidase activities of ex-PSs to those of 20S CP from PSs (Figure 5C). The T-like activities of ex-PSs and 20S CP purified from PSs were comparable (Figure 5C), consistent with a stimulatory role of the 19S complex. It is noteworthy that the CT-and C-like activities of ex-PSs were almost twice as high as those of 20S CP purified from PSs (Figure 5C).

### Identification of ex-PS subunits by MS

**Figure 6:**
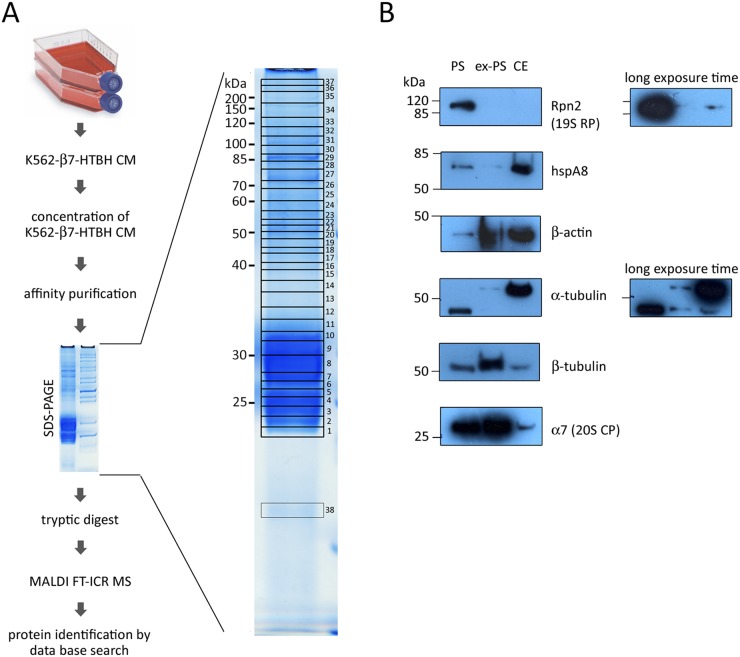
Identification of proteins associated with affinity-purified extracellular proteasomes (ex-PS) by MALDI FT-ICR MS and validation MS data of six selected proteins associated with ex-PSs and cellular proteasomes (PSs) by Western blot analysis **(A)** Culture medium (CM), conditioned by β7-HTBH K562 cells, was collected and concentrated. Proteasomes were affinity purified from the prepared conditioned medium and fractionated by SDS-PAGE. Proteins were stained with Coomassie Blue and cut into 38 pieces, which were then in-gel digested with trypsin. The peptide mixture was analyzed by MALDI FT-ICR MS. The MS data obtained were searched against the Mascot protein sequence database. **(B)** 10 μg of protein from PS, ex-PS and whole cell extract (CE) was loaded into each lane. Western blot analysis was carried out using antibodies against proteasome subunits Rpn2 and α7, hspA8, β-actin, α- and β-tubulin.

For high resolution ex-PS subunit identification by SDS-PAGE and in-gel digestion followed by MS approach, the following workflow was applied (Figure 6A). An affinity-purified ex-PS sample (15 μg) was separated by SDS-PAGE; the gel lane was sliced into 38 pieces and treated with trypsin. The generated peptides were extracted, spotted onto a MALDI target plate and analyzed (Figure 6A).

A total of 37 different proteins were identified in the purified complex by MALDI FT-ICR MS. These proteins represented either proteasome subunits (Table 1) or proteasome-interacting proteins (PIPs, Table 2). The seven β-subunits and seven α-subunits of the 20S CP appeared in multiple slices. Neither immunoproteasome subunits nor subunits of PA28 or PA200 regulatory complexes were detected. More importantly, only a few subunits of 19S RP were represented, and these by less than 8 peptides and they failed to show significant scores in the MASCOT search (Table 1). It should be noted that 6 ATPase proteins and 13 non-ATPase subunits of the 19S RP as well as PA28α, PA28γ, and PA200 have been identified previously in PSs affinity purified from K562 cells [25]. It is possible that in CM the proteasome complexes can dissociate into free 20S CP and regulatory particles due to the lack of ATP, resulting in the failure to detect regulatory subunits in affinity-purified ex-PSs by MS. This possibility is however unlikely because we were unable to detect PA200 or 19S subunits in CM at all (Figure 4C).

**Table 1:** Proteasome proteins present in purified ex-PSs and identified by MALDI FT-ICR MS

Band(s) in which detected	Proteasome subunit	Protein name	Gene name	Theoretical MW, kDa	Unique peptides	Sequence coverage, %	Delta ppm	Score
**20S proteasome**								
6, 7	α1	Proteasome subunit alpha type 6	*PSMA6*	25.4	11	33	2.26	76
4, 5, 6	α2	Proteasome subunit alpha type 2	*PSMA2*	25.8	13	59	2.07	144
8, 9	α3	Proteasome subunit alpha type 4	*PSMA4*	29.5	9	36	1.49	32
6, 7	α4	Proteasome subunit alpha type 7	*PSMA7*	27.9	13	58	2.32	93
6, 7	α5	Proteasome subunit alpha type 5	*PSMA5*	26.5	6	29	1.59	41
9, 10 11	α6	Proteasome subunit alpha type 1	*PSMA1*	29.6	11	33	2.26	165
7, 8	α7	Proteasome subunit alpha type 3	*PSMA3*	28.3	13	58	2.32	84
2, 3	β1	Proteasome subunit beta type 6	*PSMB6*	23.3	6	26	2.12	36
7, 8	β2	Proteasome subunit beta type 7	*PSMB7*	30	4	12	0.61	30
3, 4	β3	Proteasome subunit beta type 3	*PSMB3*	22.9	9	46	1.00	74
2, 3	β4	Proteasome subunit beta type 2	*PSMB2*	22.8	7	29	2.45	60
1, 2	β5	Proteasome subunit beta type 5	*PSMB5*	22.5	17	52	2.18	235
3, 4, 5	β6	Proteasome subunit beta type 1	*PSMB1*	26.5	12	52	1.43	149
5, 7, 8, 9	β7	Proteasome subunit beta type 4	*PSMB4*	24.4	7	37	1.53	66
**19S regulator particle**								
9	Rpt1	26S proteasome ATPase subunit 2	*PSMC2*	48.6	1	5	0.33	9
20	Rpt3	26S proteasome ATPase subunit 4	*PSMC4*	47.4	6	19	0.54	10
16	Rpt4	26S proteasome ATPase subunit 6	*PSMC6*	44.2	5	13	0.75	16
20	Rpt5	26S proteasome ATPase subunit 3	*PSMC3*	49.2	4	13	1.00	11
31	Rpn1	26S proteasome non-ATPase subunit 2	*PSMD2*	100.2	7	11	1.69	9
33	Rpn2	26S proteasome non-ATPase subunit 1	*PSMD1*	105.8	6	9	2.45	16
26	Rpn3	26S proteasome non-ATPase subunit	*PSMD3*	61	8	17	1.68	16
16	Rpn9	26S proteasome non-ATPase subunit 13	*PSMD13*	43	6	17	1.03	10

**Table 2:** Proteasome interacting proteins (PIPs) identified from purified ex-PSs by MALDI FT-ICR MS

Band(s) in which detected	Accession N	Protein name	Short (Alternative) name	Gene name	Theoretical MW, kDa	Unique peptides	Sequence coverage, %	Delta ppm	Score
**Chaperones**									
27	P11142	Heat shock cognate 71 kDa prote	hsp71	*HSPA8*	70.9	23	36	1.89	98
31	P07900	Heat shock protein HSP 90-alpha	hsp86	*HSP90AA1*	84.7	13	19	1.71	57
31	P08238	Heat shock protein HSP 90-beta	hsp84	*HSP90AB1*	83.3	16	25	2.10	94
28	P11021	78 kDa glucose-regulated protein	hspa5	*HSPA5*	70.2	26	45	2.17	129
38	P62937	Peptidyl-prolyl cis-trans isomerase A	cyclophilin A	*PPIA*	18.0	6	44	1.54	53
**Cytoskeleton**									
18	P60709 or P63261	Actin, cytoplasmic 1 or 2	β-actin or γ-actin	*ACTB or ACTG1*	41.7	19	51	1.71	96
22	Q9BQE3 or P68363	Tubulin alpha-1C chain or 1B chain	α -tbulin 6 or - α-tubulin ubiquitous	*TUBA1C or TUBA1B*	49.9	10	36	1.55	66
22	P07437	Tubulin beta chain	tubulin β-5	*TUBB*	49.7	18	42	2.16	132
22	Q13509	Tubulin beta-3 chain	Tubulin β-III	*TUBB3*	50.4	11	21	2.32	59
22	P68371 or P04350	Tubulin beta-4B chain or 4A chain	tubulin β-2 or tubulin 5 β	*TUBB4B or TUBB4A*	49.8	16	38	1.73	65
22	Q13885 or Q9BVA1	Tubulin beta-2A chain or 2B chain	tubulin β class IIa or tubulin β class IIb	*TUBB2A or TUBB2B*	49.9	14	33	1.75	56
**Other**									
16	P04075	Fructose-bisphosphate aldolase A	aldolase A	*ALDOA*	39.4	11	39	1.49	126
20	P06733	Alpha-enolase	enolase 1	*ENO1*	47.2	16	49	1.76	75
29, 30	P02787	Serotransferrin	transferrin	*TF*	77.1	27	28	2.17	167
35	Q00610	Clathrin heavy chain 1	CLH-17	*CLTC*	191.6	21	15	2.09	118

Fifteen of the 37 proteins identified by MS were not part of the 26S proteasome reciprocal analysis data set (Table 2). These PIPs included fructose-bisphosphate aldolase A, alpha-enolase, serotransferrin, clathrin heavy chain 1, six cytoskeleton proteins (actin and tubulins), and five chaperones. Importantly, all identified PIPs except for 3 (PPIase A, aldolase A and transferrin) have been previously shown to interact with proteasomes [24, 25, 40–45].

Additionally, to validate our findings, we performed Western blot analysis, using antibodies against a subunit of 20S CP, one of 19S RP, and the four identified PIPs, namely the chaperone hspA8 and three cytoskeleton proteins (β-actin, α- and β-tubulins). Samples of ex-PSs and PSs were normalized by total protein concentration. Overall, we observed a good correlation between the Western blot signals and the MS data (Figure 6B). All selected proteins except Rpn2 were detected in complex with ex-PSs by Western blotting.

## DISCUSSION

Since ex-PSs were detected in physiological fluids of humans and because ex-PS concentration is increased in patients with various malignancies, the possibility arose that ex-PSs are released by damage or from abnormal cells. However, poor correlation between the markers of cell death (e.g. lactate dehydrogenase release) and the level of ex-PSs has been found in other studies [46–49]. All such data exclude cell damage as a major source for ex-PSs. We have shown here that not only various human tumor cell lines, but also a primary culture of human cells release proteasome complexes (Figure 2). We have observed a positive correlation between the expression levels of PSs in tumor cells and hMESCs with the elevated ex-PS levels in CM conditioned by these cells. We also observed that, compared to tumor cells, primary cells (hMESCs) express much lower levels of PSs, which is likely to be the reason for hMESCs to release much less ex-PSs. The concentration of ex-PSs has been found elevated and correlated with disease progression for patients suffering from autoimmune diseases, multiple myeloma, lymphatic leukemia, solid tumors [4], and our results support this data. For instance, in comparison to healthy individuals, ex-PS levels were significantly increased in newly diagnosed and untreated patients with multiple myeloma [22]. In addition, patients with multiple myeloma, who positively responded to chemotherapy, had significantly lower ex-PS levels post-treatment, an effect not observed in non-responders [50, 51]. Thus, the elimination of tumor cells as a result of chemotherapy causes a decrease of ex-PS levels in physiological fluids.

Precise subunit composition and functions of ex-PSs, as well as their post-translational modifications, are still unknown. Previous studies of ex-PSs by electron microscopy showed that purified ex-PSs appear as intact 20S CPs and are proteolytically active [23]. Moreover, previously we have found by iTRAQ MS that purified ex-PSs comprise the 20S proteins together with low amounts of the 19S proteins and were able to hydrolyze fluorogenic substrates [24]. Our present data greatly substantiate these observations. Thus, according to the results of native gel electrophoresis (Figure 4B), PSs are represented by a mix of different subcomplexes, including free 20S, free 19S, singly- and doubly-capped 26S. Unlike the identified complexes of PS, ex-PS moieties are represented solely by free 20S. The lack of 19S RP subunits in the CM supports the idea that ex-PSs are released by intact cells.

In this work we followed the affinity purification strategy, using a derivative of the HTBH tag, which allowed rapid and effective purification of ex-PSs from CM conditioned by stable cell lines [25, 37]. MS analyses of the affinity-purified ex-PSs have defined 14 subunits of 20S and 8 subunits of 19S RPs. Despite the fact that some 19S subunits are defined, these failed to show reliable scores in the MASCOT search (Table 1). Western blot analysis validated the MS data and revealed in ex-PSs the 20S subunit α7, but not the 19S RP subunit Rpn2 (Figure 6B). Interestingly, neither immunoproteasome subunits nor subunits of PA28 or PA200 alternative regulator complexes were detected in ex-PSs. More importantly, 6 ATPase proteins and 13 non-ATPase subunits of the 19S RP, as well as PA28 and PA200, have been identified previously by us in PSs that were affinity purified from K562 cells [25]. Moreover, PA200 regulator has been found by iTRAQ MS in a set of the ex-PS population [24]. Because lack of ATP in CM could lead to dissociation of the proteasome complexes into free 20S and 19S (or alternative regulators), proteasome regulators might be lost from the ex-PS samples affinity purified via precipitation of individually tagged 20S subunits. To address this possibility, we verified anew the PA200 regulator by Western blotting and showed its absence in CM samples (Figure 4C). Therefore, ex-PSs consist exclusively of 20S complexes and, as a consequence, should not degrade folded proteins, because 20S substrate proteins must be at least partially unfolded to enter into the proteolytic chamber. Thus, ex-PSs may have a specific extracellular biological function associated with the inflammatory response by eliminating unfolded proteins and protein debris in extracellular space. Along with that, ex-PSs may cleave unfolded proteins, inducing antibody production, as reported in a recent study [52].

Our data indicate that the 20S CP is present and biologically active in CM, as shown by the presence of 20S proteasomal proteins in CM, by the ability of CM to hydrolyze specific proteasome fluorogenic substrates, and by their inhibition by the specific proteasome inhibitor MG132 (Figure 5). Interestingly, the CT-like proteasomal activity in CM was about 6 times lower than in CE (Figure 3B) but restored to normal levels after purification procedures (Figure 5D). It is likely that CT-like proteasomal activity in CM is inhibited by an unknown protein reversibly associated with the ex-PSs. Importantly, T-like activity of ex-PS was the same as ones of free 20S complexes uncoupled from PS (Figure 4C). However, CT- and C-like activities of purified ex-PS were about two times higher than of uncoupled 20S from PS. Similarly, C-like activity was slightly higher in CM than in CE, as opposed to down-regulated CT- and T-like activities (Figure 5D). Moreover, despite the fact that all peptidase activity levels were significantly higher in patients who had chronic lymphocytic leukemia–compared with the levels in a control group of healthy volunteers–only the C-like activity of ex-PSs was a strong predictor of survival [53]. This peptidase activity can be the main function of ex-PSs and can be regulated by specific post-translational modifications of proteasome proteins [54, 55].

Our results indicate that purified ex-PS had increased CT- and C-like activities compared with uncoupled 20S from PS. Interestingly, the immune PS has an increased CT-like activity compared with the constitutive PS [56]. It can be assumed that ex-PSs resemble immune PS. However, the immune PS has a strongly decreased C-like activity due to the replacement of β1 (C-like activity) with β1i (CT-like activity). Furthermore, we did not detect immune subunits in ex-PSs. Therefore, we believe that ex-PSs are similar to constitutive PS with increased CT- and C-like activities, which can be regulated by post-translational modifications.

It should be noted that there are some differences between the proteomic data set in our present and previous studies [24]. A small number of the 19S regulatory particle proteins, PA200 regulator, and sixty-two PIPs had been identified in complex with purified ex-PSs [24]. Here we could not detect 19S and PA200 particles in ex-PSs and identified only a set of 15 PIPs versus a set of 66 previously detected PIPs [24]. Only 6 proteins are common for both studies. Importantly, in present work we carefully controlled cell viability during cell cultivation under conditions of FBS-starvation. Hence we assumed that in our previous study ex-PSs were contaminated with intracellular proteins (including proteasomes regulatory particles 19S and PA200) because of the insufficient optimization of ex-PSs purification protocol. Furthermore, the iTRAQ proteomic approach determines the amount of the same proteins from different samples in a single experiment. So we could not reveal the distinctive features of ex-PSs versus PSs by iTRAQ MS.

One of the most important questions to be answered in the future is how the release of ex-PS is regulated, if they are transported as free 20S complexes. The activity of proteasomes is known to be regulated by post-translational modifications, such as phosphorylation, acetylation, ubiquitination, myristoylation, modification with O-linked N-acetyl-glucosamine, S-glutathionylation and oxidation, which may also influence proteasome dynamics [54, 57, 58]. For example, N-myristoylation of the 19S RP subunit Rpt2 was reported to regulate proteasome localization [59]. Possibly, ex-PSs also carry special post-translational modification(s) which regulate their release from cells. Another key question is how ex-PSs are released from cells. Virtually nothing is known about the mechanisms of ex-PS release. It is assumed that ex-PSs may leave the cell as cargo of extracellular vesicles. For example, it has been shown that microvesicles released by human primary T lymphocytes contain proteolytically active 20S CPs as well as the proteasome activator PA28 and several subunits of the 19S RP [60]. Moreover, the 20S CPs were active within exosome-like vesicles produced by human endothelial cells [52]. Proteolytically active 20S CPs have also been reported in exosomes released by mouse macrophages [61]. Our data support these results. Indeed, according to our MS data and the BRITE hierarchy search option of the KEGG database, all identified PIPs belong to the exosomal protein set (Supplementary Table 2). Consequently, it can be assumed that proteasomes acquire a specific signal due to post-translational modification(s) or by binding to some co-regulatory protein. Then a pool of free 20S particles segregates due to some specific selection or following dissociation of 26S complexes. 20S CPs need to get into lumenal vesicles (exosomes) that bud from the perimeter membrane into the multivesicular bodies (MVBs) lumen. Further MVBs fused with the plasma membrane might lead to the release of exosomes containing ex-PSs. In summary, the data presented here demonstrate that not only tumor but normal cells release ex-PS. Moreover, the population of the ex-PS does not contain regulatory particles (neither 19S RP, nor PA200) and consists exclusively of 20S complexes. Our results require further investigations into the mechanisms of ex-PS release and ex-PS functions in the extracellular space.

## MATERIALS AND METHODS

### Cell culture conditions

The human leukemia cell lines K562, KG1, THP1, multiple myeloma cell line RPMI8226, embryonic kidney cell line HEK293, and adenocarcinoma cell line HeLa were obtained from the Russian Cell Culture Collection (Institute of Cytology, St. Petersburg, Russia) where they were authenticated by STR DNA profiling analysis. The human colon carcinoma cell line HCT116 was purchased from ATCC. The cell lines K562-Rpn11-HTBH and K562-β7-HTBH, stably expressing Rpn11-HTBH [62] or β7-HTBH [25], respectively, were generated using the appropriate retroviral infection [37] and selection with puromicin. All cells were grown in RPMI 1640 or DMEM medium containing 10% (v/v) fetal bovine serum (FBS, Invitrogen) and 50 U/ml penicillin/streptomycin at 37 °C in a humidified atmosphere with 5% CO_2_. Human mesenchymal stem cells (hMESC, line 2304) were isolated from desquamated endometrium in menstrual blood and cultured in complete DMEM/F12 medium [63].

### Cell viability assays

To determine the number of viable cells present in a cell suspension, the trypan blue exclusion assay and propidium iodide (PI) flow cytometric assay were used.

To determine the extent of apoptosis, cells were harvested and co-stained with Caspase-3/7 Green Detection Reagent and SYTOX (CellEvent™ Caspase-3/7 Green Flow Cytometry Assay Kit, #C10427, Invitrogen) as recommended by the manufacturer. Thereafter, samples were analyzed with the CytoFLEX flow cytometer (Beckman Coulter) for the presence of viable (double-negative cells), early apoptotic and necrotic cells. All tests were performed in duplicate.

### Protein extraction and proteasome isolation

To prepare whole-cell extract (CE), K562 cells were lysed for 30 min at 4°C in the buffer I (50 mM Tris-HCl, pH 7.5, 100 mM NaCl, 10% glycerol, 5 mM ATP, 1 mM DTT, 5 mM MgCl_2_, 1x protease inhibitor cocktail (Roche), and 0.5% NP-40). After removal of cell debris (15,000 g, 30 min), the cell extract was assayed for protein content using a Bradford assay. Protein extracts were purified from medium (CM) conditioned by human cells K562, K562-β7-HTBH, or K562-Rpn11-HTBH. The day before CM collection, the medium from the cells was removed; cells were washed twice with PBS and cultured overnight in serum-free medium RPMI 1640 or DMEM (0.5×10^6^ cells in 1 ml of medium). On the day of collection, CM was collected, pre-cleared (300g, 10 min), centrifuged at 1200 g (10 min) and 15000 g (30 min) to remove cell debris, then concentrated (100x) using Amicon Ultra-15 filters (100K NMWL, Millipore). Subsequently, concentrated samples of CM were incubated in the buffer I for 30 min at 4°C followed disruption of extracellular vesicles by repeated freeze-thaw cycles. Proteasomes were purified from CE or concentrated CM as described previously [37]. Briefly, proteins were incubated overnight with high-capacity streptavidin-agarose beads (#20359, Thermo Scientific) at 4°C. The beads were then washed twice with 20 bed volumes of the buffer I, followed by a final wash with 10 bed volumes of TEB buffer (50 mM Tris-HCl, pH 7.5, 10% glycerol). To elute purified proteasomes, the streptavidin beads were incubated in 2 bed volumes of TEB buffer containing 1% TEV protease (T4455, Sigma Aldrich) at 30 °C for 1.5 h. The eluted proteasomes were concentrated using Amicon Ultra-0.5 filters (100K NMWL, Millipore). Approximately 10-15 μg of proteasomes is affinity purified from 0.5-1 L of medium, conditioned by K562-β7-HTBH cells overnight.

20S CPs were affinity purified from CE as described previously [64]. Briefly, purifications of 20S from PSs were performed using β7-tagged proteasomes. The protocol takes advantage of the fact that incubation of the proteasome with 500 mM NaCl caused the 20S and regulatory complexes to dissociate. When the β7 tag was used, the 20S was retained on the resin, and the non-20S subunits were eluted in the 500 mM NaCl wash step.

### Native gel electrophoresis

To resolve proteasomes from CEs and CM, native PAGE was performed on 3–12% gradient gels based on TBE buffer, as described previously [65] with some modifications. Briefly, the gel formulation was based on TBE buffer (90 mM Tris base, 80 mM boric acid, 0.1 mM EDTA, pH 8.3) containing 1 mM DTT, 1 mM ATP and 5 mM MgCl_2_. Samples were mixed with native gel loading buffer (25 mM Tris pH 8.0, 1 mM DTT, 5% glycerol, 0.01% bromphenol blue) just before loading. Electrophoresis was carried out at 6–10°C in TBE buffer containing 1 mM ATP and 5 mM MgCl_2_ at 150 V (about 30 mA) for 3 h at 4°C in a cold-room.

### Western blotting and antibodies

Proteins were separated on 12% SDS-PAGE or by native gel electrophoresis, transferred to a PVDF membrane (#1620177, 0.2 μm pore size, Bio-Rad Laboratories) and analyzed by Western blotting, using antibodies against biotin (HRP-conjugate, #7727, 1:2500, Cell Signaling Technology) and primary antibodies to α-tubulin mouse (T6074, 1:5000), β-tubulin mouse (T8535, 1:1000, all from Sigma-Aldrich), GAPDH mouse (ab9484, 1:1000), β-actin mouse (ab8226, 1:10000, all from Abcam), HSPA8/Hsc70 mouse (MABE1120, 1:500, Merck), PA200 rabbit (sc135512, 1:500, Santa Cruz Biotechnology), the subunits Rpn7 rabbit (PW8225, 1:1000), Rpn2 mouse (PW9270, 1:2000), Rpt4 mouse (PW8830, 1:1000) and α7 mouse (PW8110, 1:2000), 20S core subunits rabbit (PW8155, 1:1000, all from Enzo Life Sciences) of the 19S RP and 20S CP, respectively. Anti-rabbit or anti-mouse horseradish peroxidase (HRP)-coupled IgG secondary antibodies (Sigma Aldrich) were used, followed by ECL detection (Super Signal, Thermo Scientific).

### Assay of proteasome proteolytic activity

Chymotrypsin-, trypsin- and caspase-like (CT-, T- and C-like, respectively) peptidase activities of the proteasomes were determined using, respectively, Suc-LLVY-AMC, Ac-RLR-AMC and Z-LLE-AMC substrates (all purchased from Enzo Life Sciences) at a concentration of 0.25 mM in 50 mM Tris-HCl, pH 7.5, containing 5 mM MgCl_2_, 40 mM KCl, 1 mM DTT, 1 mM ATP at 37 °C for 45 min as described previously [66, 67]. The reaction was stopped by adding an equal volume of stop solution (0.1 M sodium chloroacetate, 30 mM sodium acetate, 25 mM acetic acid, pH 5.0). Proteasome activity was monitored by measuring free AMC fluorescence, following subtraction of background fluorescence, using a VersaFluor fluorometer (BioRad) with an excitation wavelength of 365 nm and an emission wavelength of 440 nm or a FLUOstar Omega fluorometer (BMG Labtech) with an excitation wavelength of 355 nm and an emission wavelength of 460 nm. The amount of liberated AMC was determined as fluorescence intensity. For a specificity control, the purified proteasomes were treated with 100 μM proteasome inhibitor MG132 or vehicle (DMSO).

### Preparation of samples for mass spectrometry analysis

15 μg of purified proteasomes were resolved on 12% SDS-PAGE and visualized by Coomassie staining. The protein-containing gel lane was then cut into 38 pieces and incubated twice with 60mM NH_4_HCO_3_ in 40 % acetonitrile (ACN) for 20 min at 37°C in a shaker for destaining. After drying the gel pieces with 100% ACN and vacuum evaporation they were rehydrated in 50mM NH_4_HCO_3_, 10% ACN containing 15 μg/ml proteomics grade trypsin (Sigma Aldrich) and then incubated for 30 min on ice and 4 hours at 37°C. Extraction buffer (5% formic acid/ACN, 1:2 v/v) was added to each tube and incubated for 15 min at 37°C in a shaker. Supernatant was collected, dried down in a vacuum centrifuge and dissolved in 0.1% TFA, loaded into ZipTip (Millipore), washed with 0.1% TFA and eluted with 50% ACN containing 0.1 % TFA.

### Mass spectrometry (MS)

High-resolution mass spectra were recorded on a Fourier Transform Ion Cyclotron Resonance Mass Spectrometer (Varian 902-MS) equipped with a 9.4 T magnet (FTMS) in the positive MALDI mode [68]. Samples (0.4 μl) were spotted on a steel plate with 0.4 μl of a 2,5-Dihydroxybenzoic acid matrix (Sigma Aldrich) and air-dried at room temperature and irradiated by series of 5 impulses at 355 nm from the third harmonic of a neodymium-doped yttrium aluminium garnet (Nd:YAG) laser. The laser power was set to the minimum level necessary to generate a reasonable signal. Signal from the 5 shots was recorded. A ProteoMass Peptide MALDI-MS Calibration Kit (Sigma Aldrich) was used for external calibration. For internal mass calibration, the residual trypsin peak (842.50940 Da) was used. Analysis of the MS data was carried out using FTDocViewer software (Varian) and proteins were identified using a Mascot peptide mass fingerprint software program (www.matrixscience.com). The initial search parameters allowed a mass error of up to ±2.5 ppm and a single trypsin-missed cleavage. The data sets from two MALDI FT-ICR MS were combined. Out of all identified proteins, 22 proteasome proteins and 15 proteasome-interacting proteins were identified in both biological replicates (Tables 1 and 2).

### Statistical analyses

All data are presented as mean ± standard deviation. Student's *t*-test was used for unpaired data analysis. P<0.05 was considered statistically significant.

## SUPPLEMENTARY MATERIALS FIGURES AND TABLES







## Abbreviations

ACN: acetonitrile
AMC: 7-amino-4-methylcoumarin
BSA: bovine serum albumin
CE: whole-cell extract
CM: cell conditioned medium
DMSO: dimethyl sulfoxide
DTT: dithiothreitol
ex-PS: extracellular proteasome
FBS free^*^: two-fold reduction in cell seeding density
HRP: horseradish peroxidase
HTBH tag: tag consists of two hexahistidine tags, a TEV cleavage site and a bacterially derived peptide that induces biotinylation *in vivo*
MALDI FT-ICR MS: matrix-assisted laser desorption/ionization Fourier transform ion cyclotron resonance mass spectrometry
PAGE: polyacrylamide gel electrophoresis
PBS: phosphate saline solution
PIP: proteasome interacting protein
PS: cell proteasome
SDS: sodium dodecyl sulphate
Suc: N-Succinyl
TEV: tobacco etch virus
TFA: trifluoroacetic acid.
