# Supplementary material for: Proteomic analysis of affinity-purified extracellular proteasomes reveals exclusively 20S complexes
Source: Oncotarget. 2017 Nov 1;8(60):102134–49. doi: 10.18632/oncotarget.22230 (PMC5731941; doi:10.18632/oncotarget.22230)
Supplement: Supplementary file 1 [file oncotarget-08-102134-s001.pdf]

# Proteomic analysis of affinity-purified extracellular proteasomes reveals exclusively 20S complexes

## SUPPLEMENTARY MATERIALS

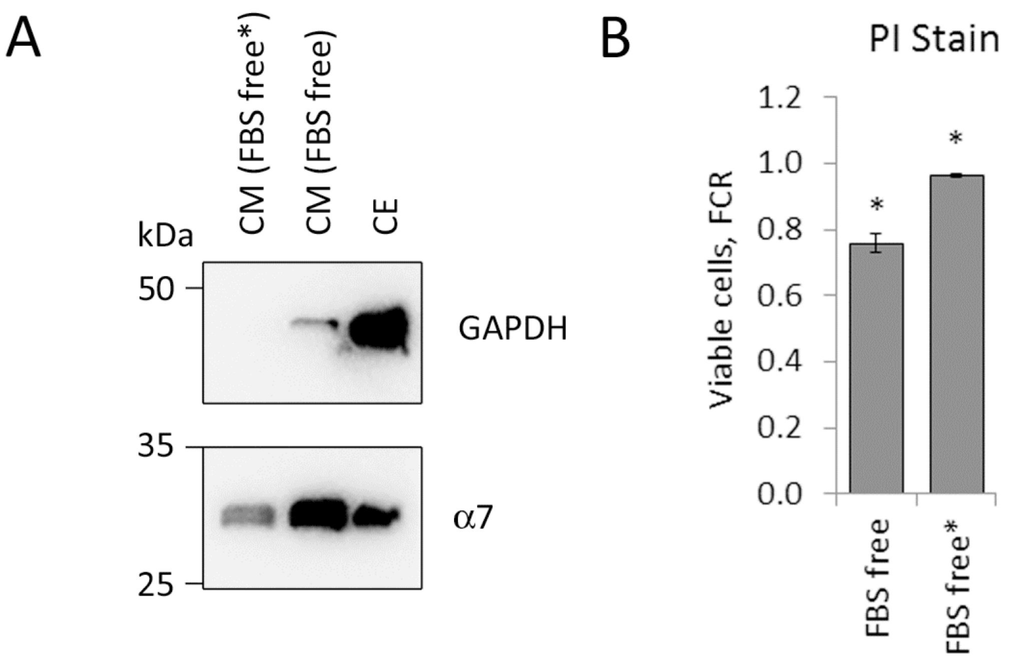


**Supplementary Figure 1: Two-fold reduction in cell seeding density (FBS free*) rescued K562 cells from serum-free (FBS free) cell death. (A)** 15 µg whole-cell extract (CE) and culture medium, conditioned by 15x$10^6$ K562 cells (CM) were subjected to SDS-PAGE and analyzed by Western blotting for their content of the cellular protein GAPDH and proteasome subunit α7. **(B)** Relative cell viability was assessed by the propidium iodide (PI) flow cytometric assay. The results are given as Fold Change Ratio relative to the cell viability of control cells (10% FBS). Values shown are mean ± standard deviation from three independent experiments (*P<0.05).

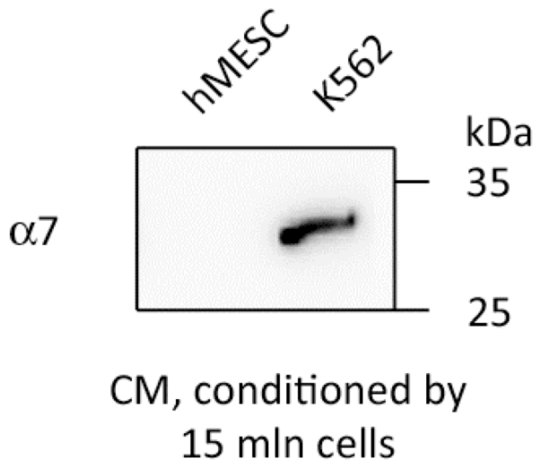


**Supplementary Figure 2: Detection of 20S proteasome subunit in CM conditioned by human K562 cells and by human mesenchymal stem cells hMESC.** Samples of CM, conditioned by 15x$10^6$ cells were subjected to SDS-PAGE and Western blotted. Representative blots of three independent experiments are shown.

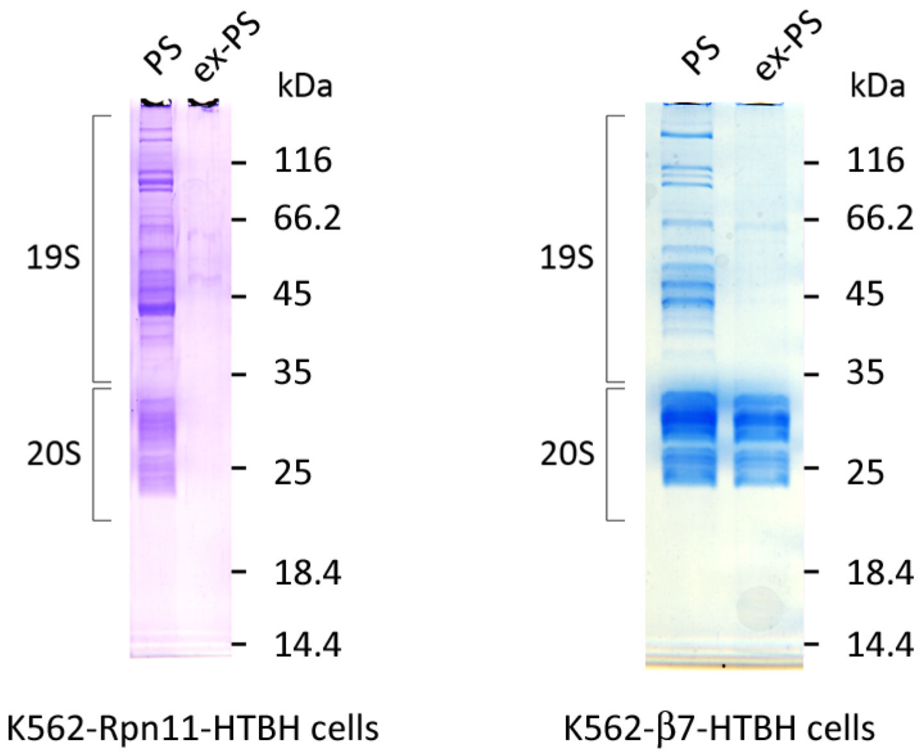


**Supplementary Figure 3: Affinity-purified proteasomes from conditioned medium of K562-Rpn11-HTBH and K562-β7-HTBH cells.** Proteins from affinity-purified cellular (PS, 10 µg) and extracellular (ex-PS, 7.5 µg) proteasomes were separated by SDS-PAGE and visualized with Coomassie Blue. Proteasomes were purified from K562-Rpn11-HTBH cells (left panel) and K562-β7-HTBH cells (right panel). Positions of 19S and 20S subcomplexes in the gel are shown.

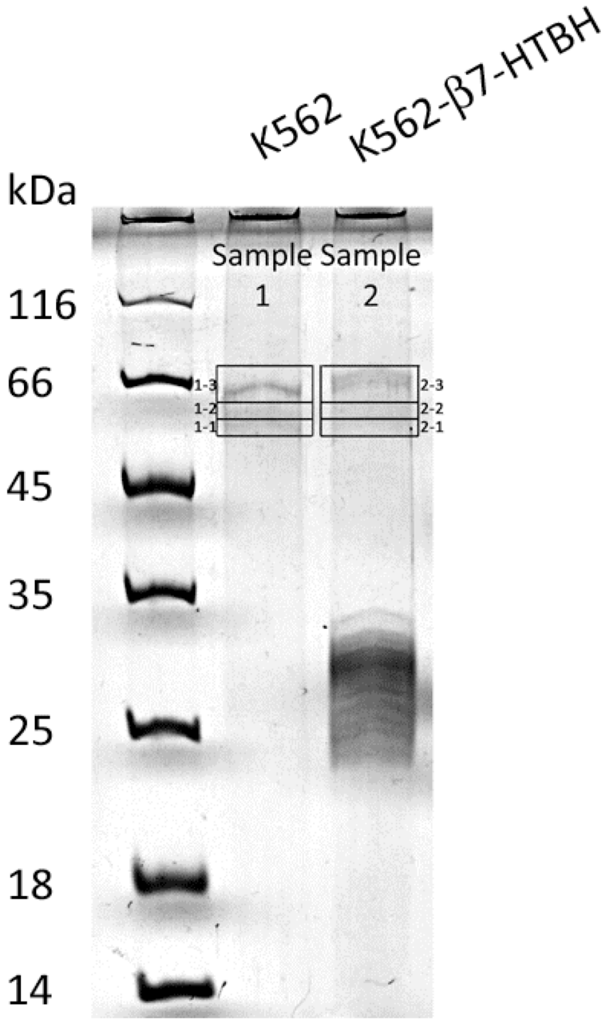


**Supplementary Figure 4: Identification of proteins associated with purified samples from conditioned medium (CM) of control K562 cells and stable cell line expressing the β7-HTBH by MALDI FT-ICR MS.** CM, conditioned by control and b7-HTBH K562 cells, was collected and concentrated. The samples were affinity purified from the prepared CM and fractionated by SDS-PAGE. Proteins were stained with Coomassie Blue and cut into 3 pieces, which were then in-gel digested with trypsin. The peptide mixture was analyzed by MALDI FT-ICR MS. The obtained MS data were analyzed using the Mascot protein sequence database.

**Supplementary Table 1: List of 1) proteins of CM K562, immobilized with streptavidin-agarose (Figure 5A and Supplementary Figure 4 and 2) proteins of CM K562-beta7-HTBH, immobilized with streptavidin-agarose (Figure 5A and Supplementary Figure 4).**

**See Supplementary File 1**

**Supplementary Table 2: Proteasome interacting proteins (PIPs) identified from purified ex-PSs by MALDI FT-ICR MS.**

**See Supplementary File 2**
