# Supplementary material for: Proteomic analysis of affinity-purified extracellular proteasomes reveals exclusively 20S complexes
Source: Oncotarget. 2017 Nov 1;8(60):102134–49. doi: 10.18632/oncotarget.22230 (PMC5731941; doi:10.18632/oncotarget.22230)
Supplement: Supplementary file 2 [file oncotarget-08-102134-s002.docx]

# Supplementary Tables

| **Supplementary Table 1. List of** | | | | | | | | | |
| --- | --- | --- | --- | --- | --- | --- | --- | --- | --- |
| **1) proteins of CM K562, immobilized with streptavidin-agarose (Fig. 5A and Suppl. Fig. 4)** | | | | | | | | | |
| **Band(s) in which detected** | **Accession N** | **Protein name** | **Short (Alternative) name** | **Gene name** | **Theoretical MW, kDa** | **Unique peptides** | **Sequence coverage, %** | **Delta ppm** | **Score** |
| 1-1 | P13645 | Keratin, type I cytoskeletal 10 | Keratin-10 | *KRT10* | 58.8 | 20 | 36 | 2 | 98 |
| 1-2, 1-3 | P35527 | Keratin, type I cytoskeletal 9 | Keratin-9 | *KRT9* | 62.1 | 22 | 60 | 2.82 | 103 |
| 1-1, 1-2, 1-3 | P04264 | Keratin, type II cytoskeletal 1 | Keratin-1 | *KRT1* | 66.0 | 30 | 47 | 2.57 | 132 |
|  |  |  |  |  |  |  |  |  |  |
| **2) proteins of CM K562-beta7-HTBH, immobilized with streptavidin-agarose (Fig. 5A and Suppl. Fig. 4)** | | | | | | | | | |
| **Band(s) in which detected** | **Accession N** | **Protein name** | **Short (Alternative) name** | **Gene name** | **Theoretical MW, kDa** | **Unique peptides** | **Sequence coverage, %** | **Delta ppm** | **Score** |
| 2-1 | P13645 | Keratin, type I cytoskeletal 10 | Keratin-10 | *KRT10* | 58.8 | 6 | 12 | 1.02 | 34 |
| 2-2 | P35527 | Keratin, type I cytoskeletal 9 | Keratin-9 | *KRT9* | 62.1 | 11 | 35 | 2.53 | 55 |
| 2-1, 2-2, 2-3 | P04264 | Keratin, type II cytoskeletal 1 | Keratin-1 | *KRT1* | 66.0 | 28 | 44 | 2.8 | 123 |
