# Supplementary material for: Proteomic analysis of affinity-purified extracellular proteasomes reveals exclusively 20S complexes
Source: Oncotarget. 2017 Nov 1;8(60):102134–49. doi: 10.18632/oncotarget.22230 (PMC5731941; doi:10.18632/oncotarget.22230)
Supplement: Supplementary file 3 [file oncotarget-08-102134-s003.docx]

| **Supplementary Table 2. Proteasome interacting proteins (PIPs) identified from purified ex-PSs by MALDI FT-ICR MS** | | | | | | | | | | | | |
| --- | --- | --- | --- | --- | --- | --- | --- | --- | --- | --- | --- | --- |
|  |  |  |  |  |  |  |  |  |  |  |  |  |
| **Band(s) in which detected** | **Protein name** | **Gene name** | **Accession N** | **Theoretical MW, kDa** | **Unique peptides** | **Sequence coverage, %** | **Delta ppm** | **Score** | **Uniprot localization** | **Kegg entry** | **Kegg pathway** | **Search BRITE hierarchies** |
| **Chaperones** | | | | | | | | | | | | |
| 27 | Heat shock cognate 71 kDa protein | HSPA8 | P11142 | 70.9 | 17 | 33 | 2.38 | 98 | Cytoplasm | hsa:3312 | Genetic Information Processing | Chaperones and folding catalysts |
|  |  |  |  |  |  |  |  |  | Cell membrane |  | Environmental Information Processing | Proteasome (Assembling factors) |
|  |  |  |  |  |  |  |  |  | Nucleus |  | Cellular Processes | Exosome (Proteins found in most exosomes) |
|  |  |  |  |  |  |  |  |  |  |  | Organismal Systems |  |
| 31 | Heat shock protein HSP 90-alpha | HSP90AA1 | P07900 | 84.7 | 13 | 19 | 1.71 | 57 | Cytoplasm | hsa:3320 | Genetic Information Processing | Chaperones and folding catalysts |
|  |  |  |  |  |  |  |  |  | Membrane |  | Environmental Information Processing | Proteasome (Assembling factors) |
|  |  |  |  |  |  |  |  |  |  |  | Organismal Systems | Exosome (Proteins found in most exosomes) |
| 31 | Heat shock protein HSP 90-beta | HSP90AB1 | P08238 | 83.3 | 16 | 25 | 2.10 | 94 | Cytoplasm | hsa:3326 | Genetic Information Processing | Chaperones and folding catalysts |
|  |  |  |  |  |  |  |  |  |  |  | Environmental Information Processing | Proteasome (Assembling factors) |
|  |  |  |  |  |  |  |  |  |  |  | Organismal Systems | Exosome (Proteins found in most exosomes) |
| 28 | 78 kDa glucose-regulated protein | HSPA5 | P11021 | 70.2 | 19 | 40 | 1.80 | 129 | Cytoplasm | hsa:3309 | Genetic Information Processing | Chaperones and folding catalysts |
|  |  |  |  |  |  |  |  |  | Endoplasmic reticulum |  | Organismal Systems | Exosome (Exosomal proteins of haemopoietic cells, colorectal cancer cells and other body fluids) |
| 38 | Peptidyl-prolyl cis-trans isomerase A | PPIA | P62937 | 18 | 6 | 44 | 1.54 | 53 | Cytoplasm | hsa:5478 | Not specified | Enzymes (Isomerases) |
|  |  |  |  |  |  |  |  |  | Secreted |  |  | Chaperones and folding catalysts |
|  |  |  |  |  |  |  |  |  |  |  |  | Exosome (Proteins found in most exosomes) |

**Table 2 (Continued )**

| **Band(s) in which detected** | **Protein name** | **Gene name** | **Accession N** | **Theoretical MW, kDa** | **Unique peptides** | **Sequence coverage, %** | **Delta ppm** | **Score** | **Uniprot localization** | **Kegg entry** | **Kegg pathway** | **Search BRITE hierarchies** |
| --- | --- | --- | --- | --- | --- | --- | --- | --- | --- | --- | --- | --- |
| **Cytoskeleton** | | | | | | | | | | | | |
| 18 | Actin, cytoplasmic 1 or 2 | ACTB or ACTG1 | P60709 or P63261 | 41.7 | 19 | 51 | 1.71 | 96 | Cytoplasm | hsa:60 or 71 | Environmental Information Processing | Cytoskeleton proteins |
|  |  |  |  |  |  |  |  |  |  |  | Cellular Processes | Exosome (Proteins found in most exosomes) |
|  |  |  |  |  |  |  |  |  |  |  | Organismal Systems |  |
| 22 | Tubulin alpha-1C chain or 1B chain | TUBA1C or TUBA1B | Q9BQE3 or P68363 | 49.9 | 10 | 36 | 1.55 | 66 | Cytoplasm | has:84790 or 10376 | Cellular Processes | Cytoskeleton proteins |
|  |  |  |  |  |  |  |  |  |  |  |  | Exosome (Exosomal proteins of haemopoietic cells, colorectal cancer cells and other body fluids) |
| 22 | Tubulin beta-4B chain or 4A chain | TUBB4B or TUBB4A | P68371 or P04350 | 49.8 | 16 | 38 | 1.73 | 65 | Cytoplasm | hsa:10383 | Cellular Processes | Cytoskeleton proteins |
|  |  |  |  |  |  |  |  |  |  |  |  | Exosome (Exosomal proteins of bladder cancer cells, colorectal cancer cells and other body fluids) |
| 22 | Tubulin beta chain | TUBB | P07437 | 49.7 | 18 | 42 | 2.16 | 132 | Cytoplasm | has:203068 | Cellular Processes | Cytoskeleton proteins |
|  |  |  |  |  |  |  |  |  |  |  |  | Exosome (Exosomal proteins of bladder cancer cells, colorectal cancer cells and other body fluids) |
| 22 | Tubulin beta-3 chain | TUBB3 | Q13509 | 50.4 | 11 | 21 | 2.32 | 59 | Cytoplasm | hsa:10381 | Cellular Processes | Cytoskeleton proteins |
|  |  |  |  |  |  |  |  |  |  |  |  | Exosome (Exosomal proteins of bladder cancer cells, colorectal cancer cells and other body fluids) |
| 22 | Tubulin beta-2A chain or 2B chain | TUBB2A or TUBB2B | Q13885 or Q9BVA1 | 49.9 | 14 | 33 | 1.75 | 56 | Cytoplasm | has:7280 or 347733 | Cellular Processes | Cytoskeleton proteins |
|  |  |  |  |  |  |  |  |  |  |  |  | Exosome (Exosomal proteins of bladder cancer cells, colorectal cancer cells and other body fluids) |
| **Other proteins** | | | | | | | | | | | | |
| 16 | Fructose-bisphosphate aldolase A | ALDOA | P04075 | 39.4 | 11 | 39 | 1.49 | 126 | Cytoplasm | hsa:226 | Metabolism | Enzymes  (Lyases) |
|  |  |  |  |  |  |  |  |  |  |  | Environmental Information Processing | Exosome (Proteins found in most exosomes) |
|  |  |  |  |  |  |  |  |  |  |  |  |  |
| 20 | Alpha-enolase | ENO1 | P06733 | 47.2 | 16 | 49 | 1.76 | 75 | Cytoplasm | has:2323 | Metabolism | Enzymes (Lyases) |
|  |  |  |  |  |  |  |  |  | Membrane |  | Genetic Information Processing | Exosome (Proteins found in most exosomes) |
|  |  |  |  |  |  |  |  |  |  |  | Environmental Information Processing |  |
| 29, 30 | Serotransferrin | TF | P02787 | 77.1 | 27 | 28 | 2.17 | 167 | Secreted | hsa:7018 | Environmental Information Processing | Not specified |
|  |  |  |  |  |  |  |  |  |  |  | Organismal Systems | Exosome (Exosomal proteins of colorectal cancer cells and other body fluids) |
| 35 | Clathrin heavy chain 1 | CLTC | Q00610 | 191.6 | 21 | 15 | 2.09 | 118 | Membrane | hsa:1213 | Cellular Processes | Not specified |
|  |  |  |  |  |  |  |  |  | Cytoplasmic vesicle |  | Organismal Systems | Exosome (Proteins found in most exosomes) |
